# Irradiation facilitates the inhibitory effect of the heat shock protein 90 inhibitor NVP-BEP800 on the proliferation of malignant glioblastoma cells through attenuation of the upregulation of heat shock protein 70

**DOI:** 10.3892/etm.2014.1800

**Published:** 2014-06-23

**Authors:** JIANYUE WU, WEIMIN WANG, QIN SHAO, GUOMIN XIAO, JUN CHENG, YUNPENG YUAN, MEI ZHANG

**Affiliations:** Department of Neurosurgery, The Affiliated Hospital of Hangzhou Normal University, Hangzhou, Zhejiang 310015, P.R. China

**Keywords:** heat shock protein 90 inhibitor, NVP-BEP800, glioblastoma, X-ray irradiation, heat shock protein 70

## Abstract

The present study aimed to investigate the effect of NVP-BEP800, a novel heat shock protein (Hsp) 90 inhibitor of the 2-aminothieno[2,3-d]pyrimidine class, in combination with radiation on glioblastoma cells. T98G human glioblastoma cells were treated with dimethyl sulfoxide (DMSO), NVP-BEP800, NVP-BEP800 in combination with X-ray irradiation (10 Gy, 20 min), or X-ray irradiation only, and cultured for 40 h. Cell viability was measured upon completion of the treatments. In addition, apoptosis was measured and immunoblot analysis was performed to analyze the expression levels of cellular protein inhibitory κB kinase β (IKKβ). The combined treatment with NVP-BEP800 and X-ray irradiation resulted in the synergistic destruction of malignant cells. Furthermore, NVP-BEP800 significantly induced apoptosis in the human glioblastoma cells. The immunoblot analysis data indicated that NVP-BEP800 markedly reduced the expression level of IKKβ. The results also revealed that X-ray irradiation significantly attenuated the increase in the level of Hsp70 in cells treated with NVP-BEP800. Since elevated levels of Hsp70 are associated with drug resistance induced by Hsp90 inhibitors, the effects of X-ray irradiation on Hsp70 levels may be associated with the enhanced effect on cells of the presence of irradiation. The results of the current study suggest that irradiation enhances the inhibitory effect of NVP-BEP800 on the proliferation of malignant glioblastoma cells by downregulating the expression level of cellular signaling protein IKKβ and attenuating the upregulation of Hsp70 that is induced by NVP-BEP800.

## Introduction

Glioblastoma is the most frequently identified type of malignant brain tumor in adults and results in serious clinical problems (1–3). Standard treatments for glioblastoma include surgery, radiation and chemotherapy. However, traditional surgery or radiotherapy alone is not fully effective and the median survival period of patients with brain cancer is not satisfactory (4,5). Radiotherapy in combination with chemotherapy has evident advantages in curing brain cancer by improving the three- to ten-year survival rates compared with those in patients treated with radiotherapy alone (6). Previous studies have investigated radiotherapy in combination with weekly nedaplatin and docetaxel chemotherapy, and indicated that chemotherapy may significantly increase the effect of radiotherapy on carcinomas, and reduce the toxicity of chemotherapy (7–9). Since chemotherapy, including nedaplatin, is toxic to normal cells, novel therapeutic agents that specifically target tumor-related cellular signaling molecules are necessary to improve treatment (6).

Heat shock proteins (Hsps) are a group of proteins that are classified according to their relative molecular masses and include Hsp10, Hsp27, Hsp40, Hsp60, Hsp70, Hsp90 and Hsp110 (10). Hsp70 has been revealed to be upregulated in certain types of cancer and may contribute to resistance to chemotherapy (11). Hsp90 is a type of chaperoning protein that is abundantly expressed in cells and is required for the expression, conformation maintenance and function of a large number of cellular proteins (12,13). Certain Hsp90-affected proteins are involved in the processes of tumor invasion, angiogenesis and metastasis (14). They are also important for the maturation and functioning of cellular signaling proteins that induce mitogen-activated protein kinases (MAPK) and nuclear factor-κB (NF-κB) pathways (15,16). Certain cellular events, including tumorigenesis, lead to the activation of the NF-κB pathway. Activation of the inhibitory κB kinase (IKK) α and IKKβ leads to kinase phosphorylation and subsequent ubiquitin-dependent degradation by the cellular proteasomal pathway (17,18). Furthermore, Hsp90 stabilizes Raf-1, Akt, and ErbB2 proteins (19–21), which are involved in the processes of that counteract radiation-induced cell death (22–24).

Inhibitors of Hsp90, including geldanamycin and its derivatives, increase the radiosensitivity of tumor cell lines derived from the glioma, prostate, pancreas and cervix. However, poor solubility, formulation difficulties and the hepatotoxicity of such compounds have limited their clinical application. Recently, the isoxazole resorcinol derivative NVP-AUY922 revealed an inhibitory effect on carcinoma cells by targeting the tumor suppressor phosphatase and tensin homolog (25). Although clinically successful in certain cancer types, one problem of Hsp90 therapy is that Hsp90 inhibitors often trigger the heat shock response, leading to an increase in the expression level of Hsp70 (26). Hsp70 induction often results in drug resistance and the advancement of the disease (27). Thus, the discovery of a method to maintain the effect of Hsp90 inhibitors without increasing the levels of Hsp70 is important.

NVP-BEP800 is a novel, fully synthetic, orally available Hsp90 inhibitor of the 2-aminothieno[2,3-d]pyrimidine class (28,29). The compound has favorable pharmaceutical and pharmacological properties. It is reported to demonstrate strong antiproliferative activity against various tumor cell lines at tolerable doses (29,30). However, the effect of NVP-BEP800 on glioblastoma remains unknown.

In the present study, the effect of NVP-BEP800 in combination with radiation on glioblastoma cells was determined. The effect of the combined treatment on cell viability and apoptosis was analyzed and the underlying mechanism was investigated. The results were evaluated to determine whether a combination of NVP-BEP800 and radiotherapy may be an effective therapeutic strategy for the treatment of glioblastoma.

## Materials and methods

### Cell culture and reagents

The T98G human glioblastoma cell line was provided by the Hangzhou Normal University (Hangzhou, China) and cultured at 37°C in a humidified 5% CO_2_ incubator. Cells were grown in Dulbecco’s modified Eagle’s medium (Invitrogen, Carlsbad, CA, USA) supplemented with 10% (v/v) fetal bovine serum (Invitrogen) and antibiotics (100 U/ml penicillin and 100 mg/ml streptomycin). NVP-BEP800 (InvivoGen, San Diego, CA, USA) were dissolved in dimethyl sulfoxide (DMSO).

### X-ray irradiation

Cells were grown in flasks and irradiated using a superficial radiotherapy system (SRT)-100 X-ray (Tema Sinergie S.p.A, Faenza RA, Italy), with a locator diameter of 10 cm, 70 kV and 10 mA, and a depth of 3 cm, at the Affiliated Hospital of Hangzhou Normal University. The irradiation (10 Gy) was performed at room temperature for 20 min. For combined treatments, cells were irradiated 24 h following the addition of NVP-BEP800 into the medium. The experiments were repeated at least three times.

### 3-(4,5-Dimethylthiazol-2-yl)-2,5-diphenyltetrazolium bromide (MTT) assay

Prior to treatments, the cells were placed into 6-well plates in medium at a density of 1×10^5^ cells/well, three wells per treatment group and cultured for 24 h. The cells were divided into treatment groups and treated accordingly for 40 h: vehicle control (DMSO, 0.016%, v/v), NVP-BEP800 (0.05, 0.1 or 0.2 μM), irradiation (10 Gy), or NVP-BEP800 (0.05, 0.1 or 0.2 μM) in combination with irradiation (10 Gy). Upon completion of the treatment, all cells were incubated with 0.5 mg/ml MTT for 3 h according to the manufacturer’s instructions (Sigma Chemical Co., St. Louis, MO, USA). The relative viability of the treated cells to the untreated control cells was measured. The absorbance was measured at 490 nm on a microplate reader (Bio-Rad Laboratories, Hercules, CA, USA).

### Apoptosis assay

Following treatment, the cells were harvested, washed twice with phosphate-buffered saline (PBS) and fixed by incubation in 4% paraformaldehyde for 30 min at room temperature. The cells were washed again with PBS to remove the fixative. The fixed cells were resuspended in PBS containing Hoechst 33258 (5 μg/ml) and incubated at room temperature for 15 min in the dark. Cells were placed on glass slides and examined for cells with apoptotic morphology (nuclear condensation and chromatin fragmentation) using a fluorescence microscope (Olympus IX81, Olympus, Tokyo, Japan) provided by Hangzhou Normal University. To determine the apoptotic level, 300 nuclei from random microscopic fields were analyzed from each sample. Data are presented as the mean percentages of apoptotic cells.

### Immunoblot analysis

The total proteins were harvested from the treated cells, separated on 10% sodium dodecyl sulfate/polyacrylamide gel electrophoresis (SDS/PAGE) gels, and then subjected to immunoblot analyses. The primary antibodies against IKKβ, Hsp70 and β-actin were purchased from Santa Cruz Biotechnology, Inc., (Santa Cruz, CA, USA); anti-IKKβ, cat# sc-8014, 1:150; anti-Hsp70, cat# sc-32239, 1:200; anti-β-actin, cat# sc-130301, 1:10,000. Secondary antibodies used in this study were goat anti-mouse immunoglobulin G (IgG)-horse radish peroxidase (HRP; cat# sc-2005, 1:5,000; Santa Cruz Biotechnology, Inc.,). Bound antibodies were detected using an enhanced chemiluminescence (ECL) system (Pierce Biotechnology, Inc., Rockford, IL, USA). The mean normalized optical density of IKKβ and Hsp70 protein bands relative to the optical density of β-actin bands from the same condition was calculated. The experiments were repeated at least three times.

### Quantitative reverse transcription-polymerase chain reaction (qPCR)

qPCR analyses of the mRNA levels of IKKβ and Hsp70 in cells were performed. The total RNAs were harvested from cells using an RNeasy kit (Qiagen, Hilden, Germany) according to the manufacturer’s instructions. The RT-PCR experiments were repeated at least three times. RNA was reverse transcribed into cDNA using random primers in an ImProm-II™ reverse transcription system (Promega Corporation, Madison, WI, USA) according to the manufacturer’s instructions. The expression levels of IKKβ and Hsp70 mRNA were quantified by qPCR using an ABI Prism^®^ 7900HT sequence detection system (Applied Biosystems, Foster City, CA, USA). The primers used are shown in Table I. An assay reagent containing premixed primers and a 4,7,2′-trichloro-7′-phenyl-6-carboxyfluorescein (VIC)-labeled probe (Applied Biosystems; cat. no. 4310884E) was applied to detect the expression levels of endogenous glyceraldehyde 3-phosphate dehydrogenase (GAPDH) mRNA. Template-negative and RT-negative conditions were used as controls. Amplification of IKKβ and Hsp70 cDNAs and the endogenous GAPDH cDNA were monitored via changes in the 6-carboxyfluorescein (FAM) and VIC fluorescent intensities, respectively, with the ABI 7900HT software (Applied Biosystems). The relative amounts of the IKKβ and Hsp70 transcripts were normalized to the amount of cellular GAPDH mRNA.

### Statistical analysis

Data are expressed as mean ± standard deviation. SPSS software, version 10.0 (SPSS. Inc., Chicago, IL, USA) was used to carry out independent sample Student’s t-tests. P<0.05 was considered to indicate a statistically significant difference.

## Results

### NVP-BEP800 in combination with X-ray irradiation inhibits the viability of glioblastoma cells

To determine whether NVP-BEP800 affects glioblastoma cells, T98G human glioblastoma cells were treated with DMSO (0.016%, v/v) only, NVP-BEP800 (0.05, 0.1 or 0.2 μM), NVP-BEP800 (0.05, 0.1 or 0.2 μM) in combination with X-ray irradiation (10 Gy, 20 min), or X-ray irradiation only (10 Gy), for 40 h. Cell viability was measured using the MTT assay following the completion of the treatments. The treatment with DMSO served as a non-drug control.

**Figure 1:**
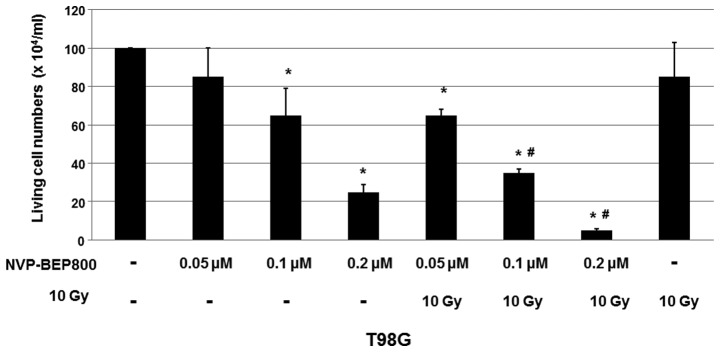
Cell viability of cells treated with dimethyl sulfoxide (DMSO), NVP-BEP800, NVP-BEP800 with X-ray irradiation, or X-ray irradiation alone. A human glioblastoma cell line, T98G, was treated with the vehicle control only (DMSO), NVP-BEP800 (0.05, 0.1 or 0.2 μM) with or without X-ray irradiation, or X-ray irradiation alone (10 Gy). Cells irradiated with X-rays were used as the irradiation control. Cell viability was measured using a 3-(4,5-dimethylthiazol-2-yl)-2,5-diphenyltetrazolium bromide (MTT) assay 40 h following the addition of NVP-BEP800. Values are expressed as means ± standard devations. ^*^P<0.05 vs. corresponding control with DMSO only; ^#^P<0.05 vs. the relative conditions in the absence of X-ray irradiation.

As shown in Fig. 1, the cell viabilities decreased by treatment with NVP-BEP800 (0.05, 0.1 or 0.2 μM) in a dose-dependent manner, when compared with the cells treated with DMSO only. Furthermore, combination with X-ray irradiation significantly enhanced the inhibitory effect of NVP-BEP800 on T98G cells, although the X-ray treatment alone only slightly reduced the cell viability. These results suggest that irradiation enhances the inhibitory effects of NVP-BEP800 on the proliferation of malignant glioblastoma cells.

### NVP-BEP800 induces apoptosis in the human glioblastoma cells

Since NVP-BEP800 reduced the proliferation of glioblastoma cells, it was investigated whether NVP-BEP800 was able to induce the apoptosis of T98G cells. Cells were treated with DMSO (0.016%, v/v) only, NVP-BEP800 (0.05, 0.1 or 0.2 μM) alone or in combination with X-ray irradiation (10 Gy, 20 min), or X-ray irradiation alone (10 Gy), for 40 h. To quantify the apoptosis, fluorescence microscopy assays were conducted following staining of the various treated cells with Hoechst 33258.

**Figure 2:**
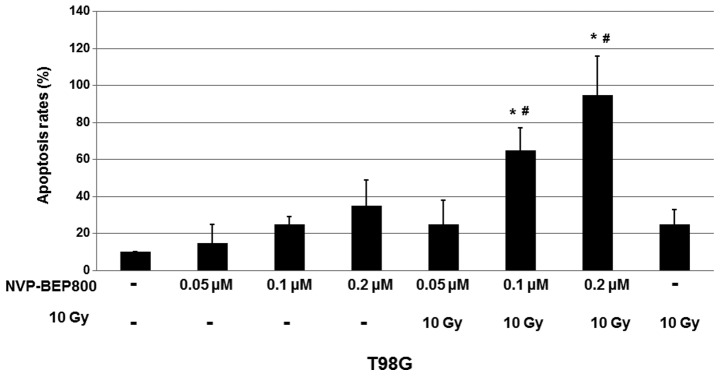
Apoptosis of cells treated with dimethyl sulfoxide (DMSO), NVP-BEP800, NVP-BEP800 with X-ray irradiation, or X-ray irradiation alone. T98G cells were treated with the vehicle control only (DMSO), NVP-BEP800 (0.05, 0.1 or 0.2 μM) with or without X-ray irradiation, or X-ray irradiation alone (10 Gy). Cells were harvested 40 h later. Hoechst 33258-stained cells were examined for apoptotic cells (nuclear margination and chromatin condensation) using a fluorescence microscope. Apoptosis rates were calculated. Data are expressed as means ± standard devations. ^*^P <0.05 vs. corresponding control with DMSO only; ^#^P<0.05 vs. the relative conditions in the absence of X-ray irradiation.

As shown in Fig. 2, treatment with NVP-BEP800 resulted in an increase in cell apoptosis. When compared with the untreated control, 0.2 μM NVP-BEP800 in combination with X-ray irradiation caused apoptosis of the T98G cells with a mean rate of ~95%. These results indicate that NVP-BEP800 combined with X-ray irradiation significantly increases the apoptosis rate of cells.

### NVP-BEP800 inhibits the expression of IKKβ protein

**Figure 3:**
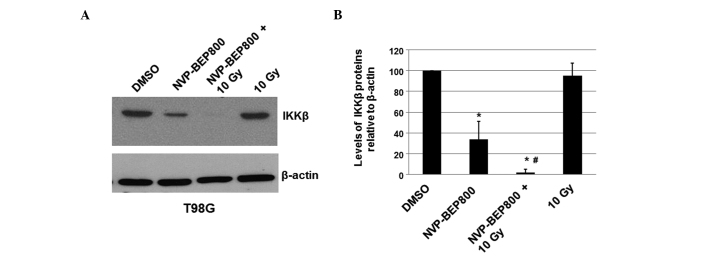
NVP-BEP800 inhibits the expression of inhibitory κB kinase β (IKKβ). T98G cells were treated with the vehicle control only (DMSO), NVP-BEP800 (0.2 μM) with or without X-ray irradiation, or X-ray irradiation alone (10 Gy). After 40 h, whole-cell extracts were isolated and immunoblot analysis was performed to analyze the expression levels of IKKβ and β-actin. The β-actin served as a loading control. (A) Representative results of immunoblot analysis. (B) Quantitative results of immunoblot analysis. ^*^P<0.05 vs. corresponding control with DMSO only; ^#^P<0.05 vs. the cells receiving X-ray irradiation only. ^*^P<0.05 vs. corresponding control with DMSO only; ^#^P<0.05 vs. the cells receiving X-ray irradiation only.

To determine whether NVP-BEP800 affects the expression level of cellular IKKβ in T98G cells, the cells were treated with vehicle control only (DMSO), NVP-BEP800 (0.2 μM) alone or in combination with X-ray irradiation (10 Gy, 20 min), or with X-ray irradiation alone (10 Gy). After 40 h, the total proteins were isolated and the expression levels of IKKβ were determined by immunoblot analysis. The cellular β-actin protein served as a loading control. The mean normalized levels of the IKKβ protein bands relative to the levels of the β-actin band under the same conditions were calculated and subjected to statistical analyses. The calculated ratios of the levels of the IKKβ proteins relative to the β-actin levels are shown in Fig. 3, along with one of the blots. The treatment of cells with NVP-BEP800 decreased the expression level of IKKβ to 28% of that in the untreated sample. These results indicate that NVP-BEP800 significantly decreases the expression level of IKKβ in the treated glioblastoma cells. Compared with cells treated with DMSO only and cells treated with X-ray irradiation only, cells treated with NVP-BEP800 and X-ray irradiation had significantly lower expression levels of IKKβ protein NVP-BEP800 and X-ray irradiation may inhibit the proliferation of glioblastoma cells through a mechanism associated with the NF-κB signaling pathway.

### NVP-BEP800 and X-ray irradiation do not affect the levels of IKKβ mRNA

**Figure 4:**
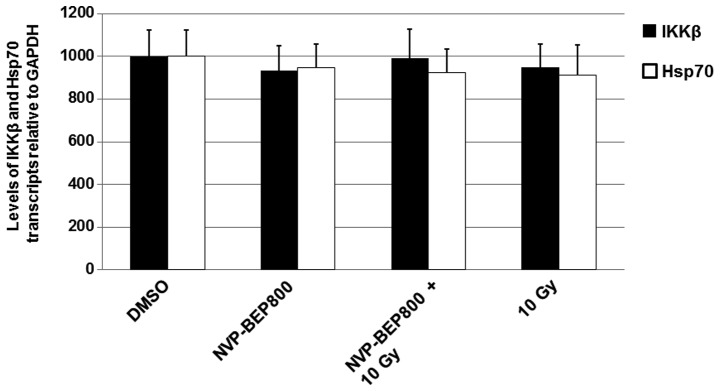
mRNA expression levels of inhibitory κB kinase β (IKKβ) and heat shock protein 70 (Hsp70). T98G cells were treated with the vehicle control only (DMSO), NVP-BEP800 (0.2 μM) with or without X-ray irradiation, or X-ray irradiation alone (10 Gy) . After 40 h, the total RNA was isolated and quantitative reverse transcription-polymerase chain reaction (qPCR) was performed to analyze the mRNA transcript levels of IKKβ and Hsp70. The level of glyceraldehyde 3-phosphate dehydrogenase (GAPDH) mRNA served as an internal control.

Changes in the levels of protein expression are often caused by altered gene transcription. Therefore, in the current study, cells were treated with DMSO, NVP-BEP800 (0.2 μM) alone or in combination with X-ray irradiation, or X-ray irradiation alone (10 Gy), for 40 h. The cells were harvested and the total RNAs were determined by qPCR. The level of mRNA transcripts in the untreated cells (DMSO) were assigned a value of 1,000. As shown in Fig. 4, the mean levels of IKKβ mRNA transcripts in cells treated with NVP-BEP800, NVP-BEP800 + X-ray irradiation, or X-ray irradiation alone were similar to those in cells treated with DMSO only. These results suggest that NVP-BEP800 and X-ray irradiation do not affect the levels of IKKβ mRNA.

### X-ray irradiation attenuates the upregulation of Hsp70 levels by NVP-BEP800

To further investigate the molecular mechanisms underlying the combined effects of NVP-BEP800 and X-ray irradiation, the possible effect of NVP-BEP800 and X-ray irradiation on the levels of Hsp70 was determined. The levels of Hsp90 were not detected as they are not detectably affected by Hsp90 inhibitors, possibly due to the high enrichment of Hsp90 in cells. T98G cells were treated with vehicle control only (DMSO), NVP-BEP800 (0.2 μM) alone or in combination with X-ray irradiation, or X-ray irradiation alone (10 Gy), for 40 h. Whole-cell extracts were isolated for the preparation of the total RNAs and proteins. qPCR was performed to detect the levels of Hsp70 mRNA. The levels of Hsp70 (Fig. 4) were not markedly altered by the treatments.

**Figure 5:**
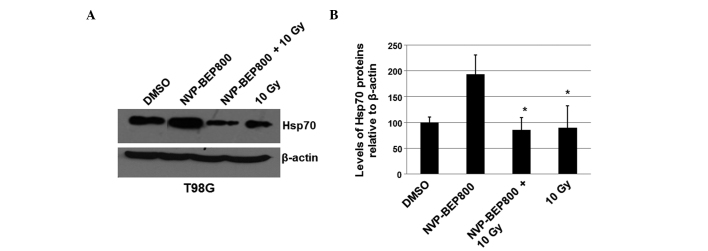
NVP-BEP800 inhibits the expression of heat shock protein 70 (Hsp70). T98G cells were treated with vehicle control only (DMSO), NVP-BEP800 (0.2 μM) with or without X-ray irradiation, or X-ray irradiation alone (10 Gy). After 40h, whole-cell extracts were isolated and an immunoblot analysis was conducted to analyze the expression levels of Hsp70 and β-actin. β-actin served as a loading control. (A) Representative results of immunoblot analysis. (B) Quantitative results of immunoblot analysis. ^*^P<0.05 vs. treatment with NVP-BEP800 (0.2 μM) alone. ^*^P<0.05 vs. treatment with NVP-BEP800 (0.2 μM) alone.

An immunoblot assay was conducted to analyze the expression level of Hsp70. It was revealed that the protein levels of Hsp70 increased in cells treated with NVP-BEP800 alone (Fig. 5). However, the increase in the levels of Hsp70 was attenuated by X-ray irradiation in the combined treatment (Fig. 5). These results suggest that X-ray irradiation may attenuate the drug resistance associated with NVP-BEP800 since the higher level of Hsp70 is associated with the drug resistance induced by Hsp90 inhibitors.

## Discussion

Standard treatments for glioblastoma include surgery, radiation and chemotherapy. Radiotherapy in combination with chemotherapy has clear advantages in curing brain cancers by improving the three to ten-year survival rate of patients compared with that in patients treated with radiotherapy alone. In the present study, the effects of NVP-BEP800 on the T98G human glioblastoma cell line were determined. NVP-BEP800 is a novel fully-synthetic, orally-available 2-amino-thieno[2,3-d]pyrimidine derivative that acts as an Hsp90 inhibitor (28–30). The current study demonstrated that combined treatment with NVP-BEP800 and X-ray irradiation resulted in the synergistic destruction of malignant cells. Furthermore, NVP-BEP800 significantly induced apoptosis in the human glioblastoma cells. These results indicate that a combined treatment with NVP-BEP800 and X-ray irradiation may be an effective strategy for the treatment of glioblastoma.

The mechanisms underlying the effects of NVP-BEP800 and X-ray irradiation were further investigated in the current study. The immunoblot analysis data indicated that NVP-BEP800 markedly reduced the expression level of the IKKβ protein. The inhibitory effect of NVP-BEP800 on the IKKβ protein may be the mechanism responsible for the effect of NVP-BEP800 on the T98G human glioblastoma cells. Since IKKβ is an important protein involved in the NF-κB pathway (15,16), it is hypothesized that the NF-κB pathway is associated with the action of NVP-BEP800. A previous study reported that NVP-BEP800 may exert a radiosensitization effect on A549 lung carcinoma and SNB19 glioblastoma cells, with a cell type-specific cytotoxicity (31). This effect may be associated with the destabilization and depletion of more than one Hsp90-affected protein (32). Multiple cellular processes may be altered through the combined use of NVP-BEP800 and X-ray irradiation, resulting in the depletion of the S phase and G2/M arrest, increased DNA damage, and the induction of apoptosis (32). All of these results suggest that NVP-BEP800 and X-ray irradiation may have important implications for tumor therapy.

Previous studies have revealed that Hsp70 is upregulated in certain types of cancer and mediates the drug resistance of Hsp90 inhibitors to chemotherapy (11,26–27). The present study demonstrated that X-ray irradiation is able to significantly attenuate the increase in the levels of Hsp70 in cells treated with NVP-BEP800. Since the higher levels of Hsp70 are associated with drug resistance to Hsp90 inhibitors (11,26,27), the effect of X-ray irradiation on Hsp70 levels may be another mechanism, in addition to the effect of NVP-BEP800 on the NF-κB signaling pathway, for the action of the combined treatment on glioblastoma cells.

## Figures and Tables

**Table I:** Primers used in the quantitative reverse transcription-polymerase chain reaction (qPCR).

Primers	Sequences (5′-3′)
IKKβ_F	5′-TGGCAATCGGCTTAGCGAT-3′
IKKβ_R	5′-GATCGGTATAGCCCGTTAA-3′
Hsp70_F	5′-CGGATTAGCCGTATGCATGC-3′
Hsp70_R	5′-GATCAATTACGGATTCGTAC-3′

IKKβ, inhibitory κB kinase β; Hsp, heat shock protein.
